# Synchronized shift of oral, faecal and urinary microbiotas in bats and natural infection dynamics during seasonal reproduction

**DOI:** 10.1098/rsos.180041

**Published:** 2018-05-02

**Authors:** Muriel Dietrich, Teresa Kearney, Ernest C. J. Seamark, Janusz T. Paweska, Wanda Markotter

**Affiliations:** 1 Centre for Viral Zoonoses, Department of Medical Virology, University of Pretoria, Pretoria, South Africa; 2 Ditsong National Museum of Natural History, Pretoria, South Africa; 3 School of Animal, Plant and Environmental Sciences, University of Witwatersrand, Johannesburg, South Africa; 4 AfricanBats NPC, Kloofsig, South Africa; 5 Wildlife Management, Mammal Research Institute, University of Pretoria, Pretoria, South Africa; 6 Centre for Emerging Zoonotic and Parasitic Diseases, National Institute for Communicable Diseases of the National Health Laboratory Service, Sandringham, South Africa

**Keywords:** bats, reproduction, microbiota, bacteria, virus, South Africa

## Abstract

Seasonal reproduction is a period of extreme physiological and behavioural changes, yet we know little about how it may affect host microbial communities (i.e. microbiota) and pathogen transmission. Here, we investigated shifts of the bacterial microbiota in saliva, urine and faeces during the seasonal reproduction of bats in South Africa, and test for an interaction in shedding patterns of both bacterial (*Leptospira*) and viral (adeno- and herpesviruses) agents. Based on a comparative approach in two cave-dwelling bat species and high-throughput sequencing of the 16S rRNA gene, we demonstrated a clear signature in microbiota changes over the reproduction season, consistent across the multiple body habitats investigated, and associated with the sex, age and reproductive condition of bats. We observed in parallel highly dynamic shedding patterns for both bacteria and viruses, but did not find a significant association between viral shedding and bacterial microbiota composition. Indeed, only *Leptospira* shedding was associated with alterations in both the diversity and composition of the urinary microbiota. These results illustrate how seasonal reproduction in bats substantially affects microbiota composition and infection dynamics, and have broad implications for the understanding of disease ecology in important reservoir hosts, such as bats.

## 1. Introduction

The microbiota is a fundamental and dynamic dimension of individuals, shaped by physiological, dietary and social factors [1], and can in turn affect host development, metabolism and susceptibility to pathogens [2,3]. Host–microbiota interactions are, thus, an important factor in vertebrate ecology and evolution [4]. However, in contrast with numerous studies on humans and laboratory animals, few studies have investigated the causes and consequences of microbiota variation in wild vertebrates, and studies based on longitudinal sampling of the same populations are especially rare [5–8].

Reproduction is a period of extreme and temporally physiological and behavioural changes [9], and has been linked to perturbation of the microbiota. For example, increased abundance of *Lactobacillus* has been observed in reproductively active females in wild mice [5]. In many animal species, seasonal reproduction is also a source of increased stress, higher contact rates and possible immunological trade-offs [10–12], which may alter host susceptibility and favour pathogen recrudescence and transmission [12,13]. Therefore, seasonal reproduction has been identified as having a significant impact on the dynamics of various host–parasite interactions, with pulses of infection being recorded, particularly in gregarious animal species [14]. In this context, microbiota perturbation during seasonal reproduction, and in relation to natural infection dynamics, could be of major importance, especially for animals that can play a significant role in human health.

Bats are well-recognized reservoirs of pathogens causing significant morbidity and mortality in humans, such as the severe acute respiratory syndrome coronavirus, as well as Marburg, Hendra and Nipah viruses [15]. New evidence suggests that bat-to-human spillovers are driven by spatio-temporal pulses of shedding in bat populations [16]. In particular, clusters of Nipah virus spillover in Bangladesh, Marburg virus in Uganda and Hendra virus in Australia have all been associated with pulses of shedding in bat colonies, specifically during the reproductive season [17–19]. Recent metagenomics-based next-generation sequencing studies have profiled the microbiota composition of bats and have shown the influence of host phylogeny, diet, body habitat and female reproductive condition [20–22]. However, to date, temporal variation of the microbiota based on longitudinal sampling has never been investigated in bats and the dynamics of the host–microbiota–infection triangle during bat reproduction thus remains poorly documented.

In this study, we investigated shifts of the microbiota in saliva, urine and faeces during seasonal bat reproduction, and its association to changes in both bacterial and viral shedding patterns. We used a comparative approach in two cave-dwelling bat species in South Africa: the insectivorous bat *Miniopterus natalensis* and the frugivorous bat *Rousettus aegyptiacus*. These species seasonally aggregate in huge numbers in maternity roosts [23,24], which make them good candidates for active transmission of infectious agents [12,13]. Indeed, they harbour several infectious agents [25–27], including some that have caused sporadic outbreaks in humans (i.e. Marburg virus) [19]. First, we used high-throughput Illumina sequencing to analyse the diversity and structure of bacterial community composition, and changes over the reproductive season. Then, we investigated the temporal dynamics of bacterial and viral shedding, and tested for any association between the observed changes in microbiota and infection dynamics. We focused on three infectious agents highly prevalent in bat populations, which are specifically excreted in three distinct body habitats: herpesviruses (HVs) in saliva, *Leptospira* bacteria in urine and adenoviruses (AdVs) in faeces. Although they may represent a risk for human health, transmission of these infectious agents from bats to humans has never been reported to date.

## 2. Material and methods

### 2.1. Field sampling

Bat sampling was conducted repeatedly over the 2015–2016 reproductive season (from September to April), in two colonies located in the Limpopo province, South Africa. We captured *M. natalensis* at Gatkop cave (S 24.61806; E 027.65223), and *R. aegyptiacus* at Matlapitsi (also known as Mahune cave) (S 24.11483; E 30.12110), 256 km from each other. These caves are maternity colonies where female bats seasonally aggregate to give birth and raise the newborns. Bats were captured using harp-traps placed at the entrance of the cave, and each individual was placed directly in a numbered cotton cloth bag. Bats were processed immediately on site using appropriate biosafety conditions, including Tyvek suits coupled with powered air-purifying respirators. Bats were morphologically identified using taxonomic keys [23,24] and we recorded the sex, the age class (adults, or juveniles = born during the 2015–2016 reproductive season) as well as the reproductive condition. We classified males as reproductive if they exhibited testicular swelling (scrotal), which is indicative of spermatogenesis. We classified females that were pregnant, post-parturient, lactating or post-lactating as reproductive, and those that were neither pregnant nor lactating by the date on which we first observed evidence of parturition as non-reproductive. Pregnant females were identified by gentle palpation of their abdomen. Recently parturient females were identified on the basis of an enlarged, less constricted and yellow vulva. We identified lactating females with enlarged nipples without fur around them. Post-lactating status was determined on the basis of enlarged dry nipples with recovering fur around them. However, fur loss from around the nipple during lactation is less in *M. natalensis* than *R. aegyptiacus*.

We collected saliva samples and, when possible, urine and faeces from the same individual. Urine and faeces were not collected for *R. aegyptiacus* as this bat species does not urinate and defecate easily during handling. Saliva was collected by carefully swabbing the tongue, the palate and the inside of the lips using sterile cotton swabs (Critical swab^®^, VWR). A urine droplet was collected using a pipet at the urethral opening. Faecal pellets were collected straight from the bats during handling, or from holding bags where the bats were kept during the capture period, using clean forceps. Each sample was placed in a sterile vial and stored in liquid nitrogen prior to being transferred to a −80°C freezer. Negative field controls (empty tubes) were included in the sampling scheme. All bats were released after sampling, except for some individuals which were euthanized by an overdose inhalation of isoflurane, and vouchers were deposited in the small mammal collection at the Ditsong National Museum of Natural History.

The sampling protocol was approved by the University of Pretoria Animal Ethics committee (EC054-14) following guidelines of the South African National Standard (SANS 10386 : 2008). Catching and collecting were carried out in strict accordance with the terms of the research permit CPM006806 and CPB6-003767 issued by the Department of Economic Development, Environment & Tourism (Limpopo province) and Section 20 approval according to the Animal Diseases Act (35 of 1984) (12/11/1/18) by the Department of Agriculture, Forestry and Fishery.

### 2.2. Sample processing and phylogeny

DNA extraction and sample preparation were performed as detailed previously [21] (electronic supplementary material, texts S1, S2, S3) after which the eluted DNA was split in two separated tubes. The first part was used for Illumina sequencing of 16S amplicons, as described previously [21]. The inclusion of negative controls during DNA extraction and library preparation was used to further identify and remove potential exogenous bacterial phylotypes following the approach of Dietrich *et al.* [21] (electronic supplementary material, text S2). The second part of samples was used for the screening of *Leptospira* bacteria based on a probe-specific real-time polymerase chain reaction [28] (later ‘RT-PCR'), and the screening of herpes- and adenoviruses using previously published nested-PCR protocols [29,30] (electronic supplementary material, text S3). The Ct of *Leptospira*-positive samples was noted to infer the relative bacterial load. Phylogenetic trees for bacterial and viral agents were constructed using BEAST [31] (electronic supplementary material, text S4).

### 2.3. Statistical analyses

All statistical analyses were conducted in R [32], primarily with the vegan, Rcmdr, MuMIn and ggplot2 packages [33–35]. We examined the temporal dynamics of bacterial and viral shedding by using generalized linear models (GLMs) with a binomial distribution and Pearson Chi-squared tests, separately for each infectious agent. Sampling period (month), sex, age class (adult/juvenile) and reproductive condition (active or not) were included as explanatory variables in the GLMs. We selected the most parsimonious model based on Akaike's information criterion. The effect of variables included in the most parsimonious model was tested using a Chi-square test (*χ*^2^) (electronic supplementary material, tables S1 and S2).

Analysis of microbiota was performed in parallel for four datasets, produced after gradual exogenous bacterial phylotype removal (electronic supplementary material, text S2). After checking for consistency among results from the different datasets, we present results for dataset D2, for which exogenous phylotypes with a relative abundance greater than 10% in the controls were removed. We analysed α- and β-diversity metrics from the rarefied phylotype tables. We calculated microbial richness using the inverse Simpson diversity index and used ANOVA to compare bacterial diversity among body habitats for *M. natalensis* (as only saliva samples were collected for *R. aegyptiacus*). Analyses of bacterial diversity were then performed for saliva, urine and faeces separately (electronic supplementary material, text S2), and the effect of the sampling period, sex, age class, reproductive condition and infection status (with HVs, AdVs and *Leptospira* bacteria) was tested using GLMs assuming a Gaussian distribution. Moreover, for urine, a Pearson's correlation test was performed to analyse the microbiota diversity in relation to the *Leptospira* load (cycle threshold value measured by RT-PCR).

To analyse microbiota composition, non-metric multidimensional scaling ordinations were conducted on Bray–Curtis dissimilarities, calculated from rarefied sequence counts, after square root transformation and Wisconsin standardization. For each infectious agent, separate permutational MANOVA (PERMANOVA) tests with 999 permutations were performed to test the microbiota structure between sampling months, sexes, reproductive conditions and infection status. We then used linear discriminant analysis effect size (LEfSe; Galaxy v. 1.0) to identify phylotypes that differed significantly in relative abundance between the identified factors. We set the α value for the Kruskal--Wallis test at 0.05 and the threshold on the logarithmic LDA score at 2.0.

## 3. Results and discussion

### 3.1. Bat samples and population dynamics

**Figure 1.**
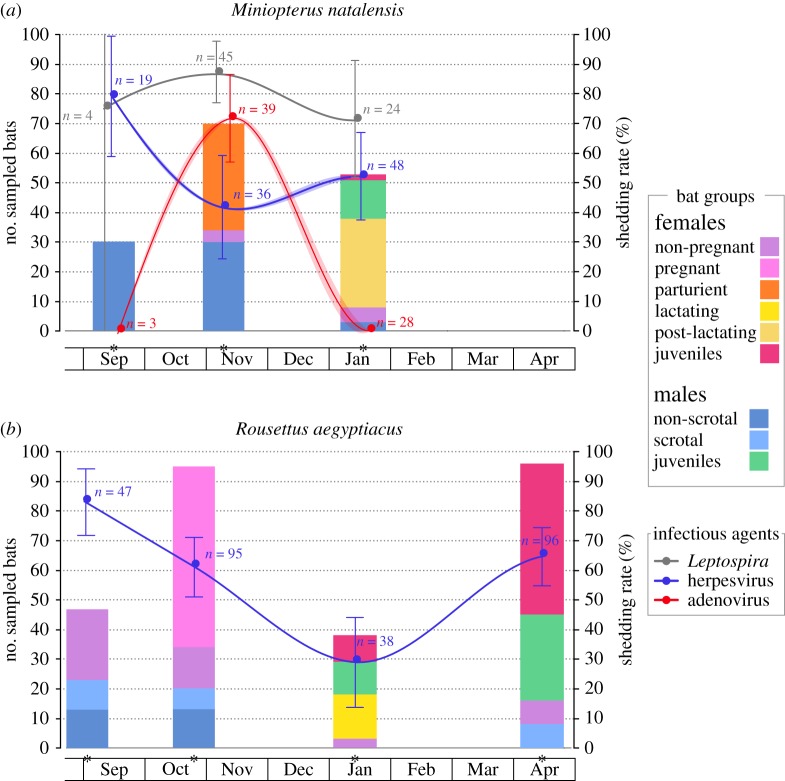
Distribution of bat groups according to sex, age class and reproductive stages, and temporal infection dynamics in two maternity colonies of (*a*) *M. natalensis* and (*b*) *R. aegyptiacus*. Sampling sessions are denoted by a star on the *x*-axis. The continuous lines represent the prevalence of infections modelled by GLM and the shaded area the 95% confidence interval. Curves were produced using a loess smoother. Observed prevalence rates at each sampling session are represented by dots with the 95% confidence interval and the number of tested samples indicated. Elements have been moved slightly on the *x*-axis to avoid superimposition.

A total of 542 samples were collected longitudinally during the 2015–2016 reproductive season (electronic supplementary material, table S3). Samples were obtained from three separate sessions (September 2015, October/November 2015, January 2016) for both bat species, and an additional session (April 2016) was conducted for *R. aegyptiacus* (figure 1). In total, 103 and 276 saliva samples were, respectively, obtained for *M. natalensis* and *R. aegyptiacus*. In addition, 73 urine and 90 faecal samples were collected for *M. natalensis*.

In September 2015, we trapped exclusively non-scrotal males in the *M. natalensis* colony, while in the *R. aegyptiacus* colony, there were both non-scrotal and scrotal males, along with non-pregnant females (figure 1). In November 2015, most (90%) of the females sampled in the *M. natalensis* colony were parturient (i.e. had given birth very recently; pregnant females were released without sampling) and the males were again all non-scrotal. For *R. aegyptiacus*, the sampling performed in October 2015 comprised a majority (64%) of pregnant females, but also non-pregnant females, and both scrotal and non-scrotal males. In January 2016, large amounts of juveniles were present in both colonies, with mainly post-lactating females for *M. natalensis* and lactating females for *R. aegyptiacus*. Finally, in April 2016, most of the bats caught in the *R. aegyptiacus* colony (83%) were flying juveniles.

### 3.2. Temporal dynamics and genetic diversity of infectious agents

**Figure 2.**
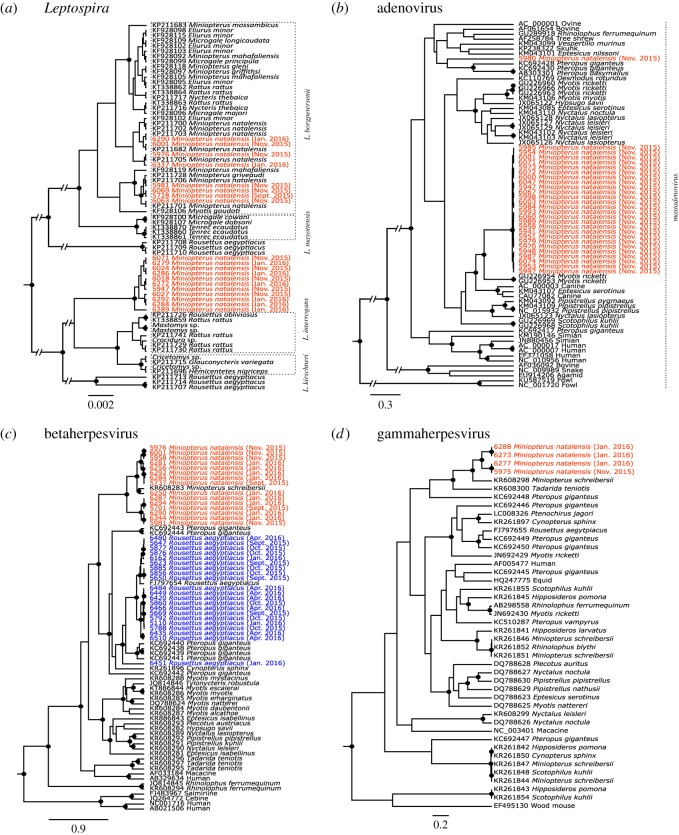
Phylogenetic relationships of (*a*) *Leptospira*, (*b*) adenovirus and (*c*,*d*) herpesvirus detected in *M. natalensis* and *R. aegyptiacus* colonies. Samples from this study are represented in red and blue, respectively, for *M. natalensis* and *R. aegyptiacus*, and are coded with sample ID, host species and date of sampling. Posterior values higher than 75% are represented by a dark circle at the nodes.

For *M. natalensis*, among the bats sampled at two (*n* = 85) or three (*n* = 30) body habitats, 45% were infected by two infectious agents and 17% were infected by the three infectious agents. *Leptospira*-positive urine samples in *M. natalensis* clustered in two genetic groups (figure 2*a*), one associated with *L. borgpetersenii* samples previously identified in *M. natalensis* in South Africa [26] and the other closely related to *L. interrogans* and *L. kirschneri*. High *Leptospira* shedding prevalence was observed across the reproductive season, varying between 71% (±18%) and 87% (±10%). Despite some variation over time and a peak in November that coincided with parturition (figure 1*a*), we found no significant effect of the sampling month for *Leptospira* shedding prevalence in urine. This may be due to a lack in statistical power, because of the limited number of urine samples available in September (*n* = 4). Only a significant effect of age class on *Leptospira* shedding prevalence was found (GLM_1_: $\chi_{1}^{2}=6.851$, *p* = 0.009; electronic supplementary material, table S1), but this was because none of the two urine samples collected from juveniles were positive for *Leptospira*, compared to an overall shedding prevalence of 83% (±9%) in adults. Our results are consistent with previous studies reporting bats as a reservoir of pathogenic *Leptospira* worldwide [36], and similar to the dynamic found in the insectivorous bat *Mormopterus francoismoutoui* in Reunion Island, where a peak of shedding was observed in late-pregnant females [12].

AdVs detected in faeces of *M. natalensis* were almost all identical and closely related to mastadenoviruses found in the bat *Myotis ricketti* in China [30]. One sample (UP5980) clustered with a sequence from the fruit bat *Pteropus giganteus* in Bangladesh (figure 2*b*). We found a strong variation of AdV shedding prevalence over the reproductive season (GLM_2_: $\chi_{2}^{2}=65.197$, *p* < 0.001), as shedding only occurred in November with 72% (±14%) of the bats excreting AdV (figure 1*a*). Our limited number of faeces samples collected in September (*n* = 3) may hide a higher prevalence in the *M. natalensis* colony, but results from January (*n* = 28 samples) showed that lactating and post-lactating females, as well as young juveniles, were not shedding AdV. This contrasts with the stable prevalence observed by Drexler *et al.* [13] in *Myotis myotis* in Germany, before and after parturition. The absence of AdV shedding in young juveniles may be explained by the transfer of maternal antibodies, as demonstrated experimentally for henipaviruses [37,38] and largely suggested by several field studies [12,13,39,40]. However, the absence of AdV shedding in lactating and post-lactating females suggests that even though persistence of DNA viruses on the level of individual bats might occur [13], shedding is highly dynamic over the reproduction period in female individuals.

Phylogenetic analysis of saliva samples showed that the *M. natalensis* colony was infected with β- and γ-HVs (figure 2*c,d*), both genetically related to sequences detected in *Miniopterus schreibersii* in Spain [41]. By contrast, only β-HVs were detected in *R. aegyptiacus* (figure 2*c*). Sequences were embedded within a clade containing other fruit bat samples and were closely related to the only sequence available for *R. aegyptiacus*, obtained from a captive bat at the Budapest zoo in Hungary [42]. For both bat species, HV shedding prevalence varied strongly over time (GLM_3_: $\chi_{2}^{2}=8.848$, *p* = 0.012, GLM_4_: $\chi_{3}^{2}=36.364$, *p* < 0.001, figure 1*b*) and with age class (GLM_3_: $\chi_{1}^{2}=12.169$, *p* < 0.001, GLM_4_: $\chi_{1}^{2}=3.913$, *p* = 0.048). However, we found a distinct temporal pattern compared to *Leptospira* and AdV. Indeed, a peak of HV shedding was observed at the beginning of the reproductive season, followed by an almost twofold decrease in November for *M. natalensis* and a continuous decrease in October and January for *R. aegyptiacus*. The effect of age class was explained by the lower shedding prevalence that was observed in juveniles, suggesting the protection of juveniles by maternal antibodies during the first months of life. Additional sampling of *R. aegyptiacus* in April allowed us to observe a second peak of shedding (65 ± 10%), which may reflect the progressive loss of maternal protection and thus the infection of older juveniles. We also found that reproductive status was associated with an increase of HV shedding prevalence in *R. aegyptiacus* (GLM_4_: $\chi_{1}^{2}=6.028$, *p* = 0.014), but this was probably driven by the absence of HV shedding in juveniles (thus in a non-reproductive state) in January compared to a shedding prevalence of 67% (± 24%) in lactating females at the same time ($\chi_{1}^{2}=16.944$, *p* < 0.001). In females, pregnancy and lactating states were not associated to a different HV shedding prevalence compared to non-reproductive females. In males, although scrotal individuals always showed a higher HV shedding prevalence (100% in September, 85% in November) compared to the non-scrotal ones (77% and 46%, respectively), differences were not statistically significant. Overall, our results illustrate the HV temporal dynamics in bats, with similarity between two bat species. A notable aspect of HV infection is its latency, despite ongoing immunity, combined with the capacity to reactivate and spread to new hosts, especially during stress and pregnancy [43–45]. Moreover, male reproductive behaviours (such as biting) could be an important component of natural HV transmission, as shown in mice [46,47]. Therefore, we could hypothesize that such factors may play an important role in favouring HV transmission at the beginning of the bat reproductive season.

### 3.3. Variation in microbiota diversity

**Figure 3.**
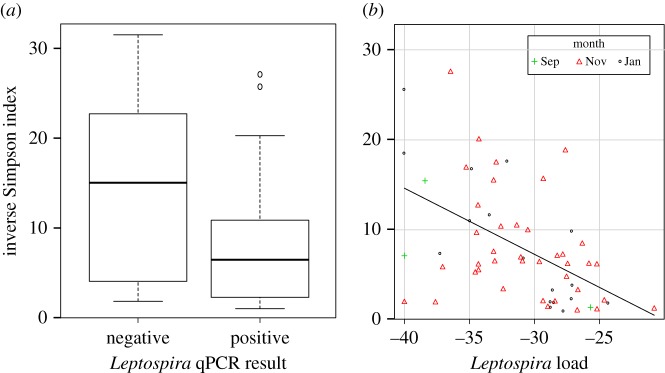
Decrease of microbiota diversity in urine of *M. natalensis* associated with *Leptospira* shedding. In (*b*), *Leptospira* load is represented by the mean Ct values (in negative form) for qPCR-positive *Leptospira* samples.

Based on the analysis of the Illumina-sequenced 16S amplicons, we found a significant change of bacterial community diversity over the reproductive season in the oral microbiota only, for both bat species (GLM_7_: $\chi_{2}^{2}=1562.4$, *p* < 0.001; GLM_8_: $\chi_{3}^{2}=5.245$, *p* = 0.004) (electronic supplementary material, table S1). In *M. natalensis* for example, bacterial diversity in saliva was particularly high in September (electronic supplementary material, figure S1*a*). In faeces, bacterial community diversity was driven by sex (GLM_6_: $\chi_{1}^{2}=36.123$, *p* = 0.002), with females harbouring less diversity compared to males (electronic supplementary material, figure S1*b)*. This may be explained by a confounding effect of reproductive status; as in *M. natalensis*, all males were in a non-reproductive state (non-scrotal) and most females were reproductively active. This would contrast with the results of Phillips *et al.* [20] from phyllostomid bats from South America, where reproductive females had the highest microbiota diversity compared to all other bat groups (scrotal males, non-reproductive males and non-reproductive females). A marginal difference between sexes was also observed in the saliva of *R. aegyptiacus* (GLM_8_: $\chi_{1}^{2}=1.651$, *p* = 0.040), but with the opposite pattern (electronic supplementary material, figure S1*b*). Finally, we did not find any association between infectious agent shedding and the diversity of bacterial communities in the respective body habitats, except for *Leptospira* bacteria in urine. Indeed, the presence of *Leptospira* bacteria (detected by RT-PCR) in urine in *M. natalensis* was associated with lower diversity in the urinary microbiota (GLM_5_: $\chi_{1}^{2}=447.320$, *p* = 0.004, figure 3*a*), and as expected the urinary microbiota diversity decreased with increased *Leptospira* load (*r*^2^ = 0.52, *p* < 0.001, figure 3*b*).

### 3.4. Drivers of microbiota composition

**Figure 4.**
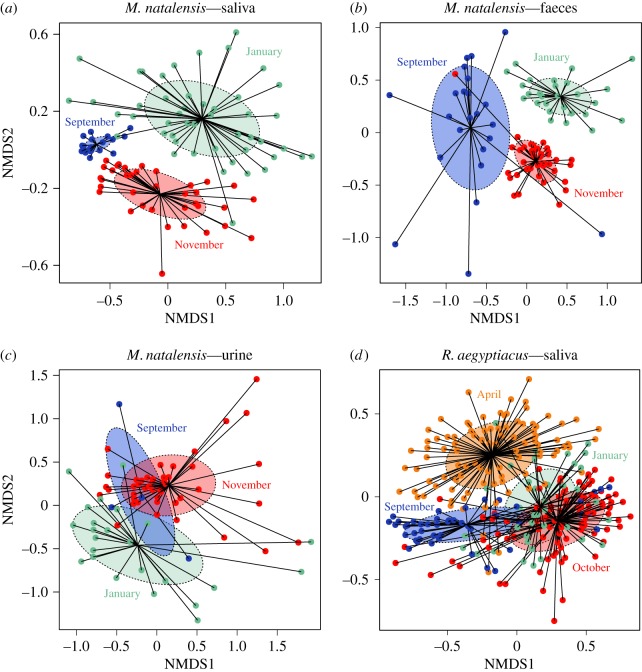
Shift of microbiota composition over the reproductive season in (*a–c*) *M. natalensis* and (*d*) *R. aegyptiacus*. Each data point represents the microbiota from one bat. Shaded ellipses represent one standard deviation around sample group centroids.

We observed a strong variation of microbiota composition over the reproductive season, in the three body habitats (PERMANOVA: all *p* = 0.001, figure 4). In saliva and faeces of *M. natalensis*, microbiota composition was also strongly dependent on the sex (PERMANOVAs: both *p *= 0.001). Indeed, LEfSe analysis revealed that most phylotypes were enriched in males, although a few were significantly more abundant in females, both in saliva and faeces, such as members of Pasteurellaceae, *Gemella* and *Haemophilus*. This could be explained by endocrine/steroid differences, as already suggested for humans [48], but also potential differences in feeding behaviours between males and females [49].

A significant effect of reproductive condition was found in the saliva of *R. aegyptiacus*, with the composition of oral microbiota being different between pregnant and non-pregnant adult females (PERMANOVA: *p* = 0.026). For example, LEfSe analysis revealed that salivary microbiota of pregnant females was enriched with *Actinobacillus* and *Streptococcus*. In contrast, no significant difference was observed in the saliva of *M. natalensis* females (analyses were not performed for faeces and urine because of the limited sample size for non-pregnant females). The effect of reproductive condition in males was tested in *R. aegyptiacus* only, as no scrotal males were caught in the *M. natalensis* colony, but we found no significant difference of salivary microbiota composition between scrotal and non-scrotal adult males (PERMANOVA: *p *= 0.707). Such a relationship between female reproductive condition and microbiota composition has already been reported in South American bats [20], and is consistent with findings in humans showing that the gut microbiota is altered by pregnancy [50].

**Figure 5.**
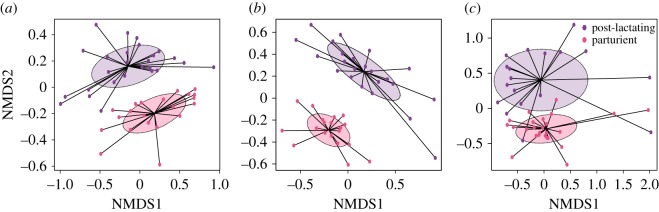
Shift of microbiota composition in reproductive females of *M. natalensis* in (*a*) saliva, (*b*) faeces and (*c*) urine. Each data point represents the microbiota from one bat. Shaded ellipses represent standard deviation around sample group centroids. Parturient and post-lactating females are denoted by different colours.

Shift of microbiota composition in reproductive females was evidenced in *M. natalensis*, in which, parturient and post-lactating females had completely distinct microbiotas for the three body habitats (PERMANOVAs: all *p *< 0.01, figure 5). Indeed, microbiota of parturient females were enriched in *Staphylococcus* and *Corynebacterium* in all body habitats, while diverse phylotypes were associated with the post-lactating status, such as *Streptococcus* in faeces, *Haemophilus* in saliva and members of Planctomycetaceae in urine. It is likely that the overall immune modulation during pregnancy plays a role in alteration of the microbiota [51]. Surprisingly, in *R. aegyptiacus*, no significant difference was found in salivary microbiota composition between pregnant and lactating females (PERMANOVA: *p* = 0.75). One explanation could be that the two bat species were not sampled at exactly the same reproductive stages and that shift of microbiota was probably stage-dependent. For example, Collado *et al.* [52] have reported extreme changes in microbiota from the first to the third trimester of pregnancy in women, while others have shown some stability over the perinatal period [53]. Therefore, it is crucial to consider the time frame and reproductive stages sampled. Overall, our results showed that a general shift in microbiota composition may occur in reproductive female bats, but further studies are needed to determine whether the processes are similar to what is observed in humans [51].

We did not find any association between HV shedding in saliva and associated bacterial microbiota for both bat species (both PERMANOVAs: *p* > 0.05). By contrast, we found a significant association between AdV presence and faecal microbiota composition in *M. natalensis* (PERMANOVA: *p* = 0.001), but this coincided with the effect of sampling session, as bats were found shedding AdVs only in November. Therefore, it is difficult to conclude if there was an interaction between AdV shedding in faeces and the faecal microbiota composition. Finally, as noted with the urinary microbiota diversity, we also found a strong correlation between *Leptospira* shedding (measured by RT-PCR) and urinary microbiota composition in *M. natalensis* (PERMANOVA: *p* = 0.001). LEfSe analysis confirmed that *Leptospira* RT-PCR positive samples were enriched in *Leptospira* but also in *Illumatobacter*, while 31 other phylotypes, including *Clostridium sensu stricto* and unclassified Bacillales, were predominantly associated with negative samples. Altogether, our results suggested no interaction between DNA virus shedding dynamics and shift of bacterial microbiota in bats. To our knowledge, interactions between virus dynamics and microbiota have never been demonstrated in wild animals, but recent field studies have reported an association between microbiota composition and other types of parasites, such as in frogs naturally infected by the chytrid fungus [54], rodents parasitized by multiple helminths [55] or even bats for which invasion by the fungus *Pseudogymnoascus destructans* leads to a shift in the skin microbiota [56]. Moreover, experimental studies performed in mice, using RNA viruses, have shown interactions with the microbiota, through diverse effects such as pathogen inhibition, co-aggregation and regulation of the immune system and metabolism [2]. Our lack of interaction for herpes- and adenoviruses may be due to the fact that other aspects of the microbiome (other than bacteria) could interact with viral infection. These viruses are also both DNA viruses capable of latency and reactivation at the individual level, and thus may be more involved in long-lasting changes of the microbiota and the modulation of immune functions [57]. Further investigations, targeting virus pool dynamics in bats and specifically RNA viruses, will assist in determining how transient and chronic infections may interact with the dynamics of microbiota composition.

## 4. Conclusion

Based on a unique longitudinal sampling of multiple body habitats and a comparative approach on two bat species, we investigated the within-colony dynamics of both microbiota and viral/bacterial shedding during the seasonal reproduction of bats. Our results demonstrated that seasonal reproduction is characterized by substantial changes of the microbiota and shedding patterns, which are mainly driven by sex, age and reproductive condition of female bats. This study illustrates the complexity of host–microbiota–infection interactions in a significant group of mammals, which are also considered as important hosts of human pathogens with seasonal dynamics (e.g. Marburg and Hendra viruses). Our findings thus provide interesting evidence as to how pathogens may shift seasonally due to life-history events that may alter the microbiota and immune system of their hosts, which in turn could have important public health consequences.

## Supplementary Material

Supplementary Material from “Synchronized shift of oral, fecal and urinary microbiotas in bats and natural infection dynamics during seasonal reproduction”

## Data accessibility

The list of samples, the Illumina reads of the 16S bacterial sequence data and the OTU tables have been deposited in Dryad (http://dx.doi.org/10.5061/dryad.54f1jc2) [58]. Genetic sequences generated for *Leptospira*, herpesvirus and adenoviruses, using specific primers, are available on GenBank under accession numbers MG680317–MG608402.
