# Supplementary material for: Synchronized shift of oral, faecal and urinary microbiotas in bats and natural infection dynamics during seasonal reproduction
Source: R Soc Open Sci. 2018 May 2;5(5):180041. doi: 10.1098/rsos.180041 (PMC5990816; doi:10.1098/rsos.180041)
Supplement: Supplementary Material from “Synchronized shift of oral, fecal and urinary microbiotas in bats and natural infection dynamics during seasonal reproduction” [file rsos180041supp1.pdf]

Supplementary Material

Synchronized shift of urine, faeces and saliva microbiotas in bats and natural infection dynamics during seasonal reproduction

Muriel Dietrich, Teresa Kearney, Ernest C. J. Seamark, Janusz T. Paweska and Wanda Markotter

ROYAL SOCIETY OPEN SCIENCE

Text S1. DNA extraction protocol

Text S2. Illumina sequencing and bioinformatics

Text S3. Analysis of bacterial and viral shedding

Text S4. Phylogenetic analyses of infectious agents

Table S1. Summary of statistical models used to analyse infection dynamic and microbiota diversity

Table S2. Model selection tables.

Table S3. Details of the number of bat samples collected at different sample periods

Figure S1. Variation of microbiota diversity

References

Text S1. DNA extraction protocol

We used a previously validated DNA extraction protocol developed for the Human Microbiome Project [1,2], recently used for bat samples [3]. Care was taken to avoid bacterial DNA contamination by utilizing DNA-free reagents when applicable, filter sterilizing all solutions through a 0.2 µM filter, and working in a PCR-clean hood. Before DNA extraction, faeces samples were weighted and the volume of urine samples was measured. To control for the introduction of contaminating DNA, negative extraction controls (reagents only) were included in the extraction procedure. The protocol includes the addition of the peptidoglycan-degrading enzymes mutanolysin and lysozyme and the use of the QIAmp DNA Micro kit (Qiagen, Valencia, CA), as previously described [3]. We used a 3 hours incubation time at 56°C, the addition of carrier RNA, and faeces samples were centrifuged (2 min. at 604*g*) and the supernatant collected before the transfer to the Qiagen column. DNA was eluted into 32 µl of buffer AE and stored at -20°C.

Text S2. Illumina sequencing and bioinformatics

We subjected the DNA extracts and the negative controls (from the field and the extraction) to V3-V4 region 16S rRNA PCR and barcoded Illumina sequencing, following the standardized and optimized Metabiote® protocol developed by Genoscreen (Lille, France). Samples from *M. natalensis* and *R. aegyptiacus* were processed separately, with the production of two distinct libraries. Additional negative and positive PCR controls, in the form of an ultrapure water sample and a mock community respectively, were provided by Genoscreen and included in the preparation of each library. The indexed DNA libraries were equimolar pooled and diluted at a final concentration of 4 nM. Paired-end (2 x 250) sequencing was performed using a MiSeq Reagent Nano kit on two different Illumina MiSeq runs, using two lanes each, using PhiX DNA (15%) as a spike-in control for the estimation of the error rate during sequencing.

Libraries were de-multiplexed using Casava version 1.8 and raw reads were recovered as FASTQ files. Forward and reverse primers were removed at 100% nucleotide identity by Genoscreen and sequences were quality trimmed when Q score < 30. Alignment of PhiX reads to the reference genome yielded an error rate during sequencing comprised between 1.13-1.42% for read 1 and 1.10-1.33% for read 2 (depending on the lane). The correct taxonomic assignation of the 11 bacterial species present in the positive control was validated by Genoscreen, using the Greengenes reference database (DeSantis *et al.* 2006). Samples were then analyzed using MOTHUR v.1.33.3 following the MiSeq SOP Pipeline [4,5]. Assembled reads were quality trimmed based on their length prior to alignment against the MOTHUR-formatted SILVA database. Preclustering of the data was performed using a 4-bp difference, following by the detection and removal of chimeras using the UCHIME algorithm [6]. We then classified sequences using the MOTHUR-formatted version of the RDP training set (v.9), and any unknown, chloroplast, mitochondrial, archaeal, or eukaryotic sequences were removed. Remaining sequences were clustered into phylotypes using a 97% identity threshold.

Using both the negative and positive controls, we identified potential exogenous DNA from laboratory reagents, as previously described from low-biomass bat samples [3]. We therefore produced, for each bat species, different datasets corresponding to different levels of exogenous phylotypes removal, following the approach described in Dietrich *et al.* [3]. Briefly, in the first dataset (D1) all phylotypes were included; in the second (D2) and third (D3) datasets, exogenous phylotypes with a relative abundance > 10% and > 0.1% in the controls respectively were removed; and in the fourth dataset (D4), all exogenous phylotypes were removed.

Preliminary analyses confirmed that both bacterial community diversity (ANOVA: $p = 0.001$) and composition (PERMANOVA: $p = 0.001$) were different among body habitats in *M. natalensis* [3]. Therefore, we analyzed separately the microbiota of each body habitat.

Text S3. Analysis of bacterial and viral shedding

The second half of DNA extracts from saliva and faeces samples were used as templates for herpesvirus and adenovirus detection respectively, using previously published nested-PCR protocols both targeting the DNA polymerase gene [7,8]. Briefly, both PCRs were ran in 25µl total volume containing 12.5µl of DreamTaq Green PCR Master Mix (Thermo Fisher Scientific) and 1 µM of each primer. After electrophoresis on a 2% agarose gel, PCR products of the approximate anticipated size were submitted to Sanger sequencing (Inqaba). For the detection of *Leptospira* bacteria in urine samples, a probe-specific real-time PCR, targeting the 16S rRNA gene, was performed as previously described [9]. All PCRs were ran with a negative (PCR mix only) and positive controls (i.e. DNA from a Human herpesvirus 1 cell culture, DNA from an adenovirus-positive bat (*Neoromicia* spp.) sample, DNA from a *Leptospira interrogans* culture). For *Leptospira* positive samples, a partial fragment of the 16S rRNA gene was then amplified, as described in Dietrich *et al.* [10] and sequenced.

Text S4. Phylogenetic analyses of infectious agents

Nucleotide sequences of *Leptospira*, adenoviruses and herpesviruses were checked individually with ChromasLite 2.01 (Technelysium Pty, South Brisbane, Australia) and then assembled and aligned with reference sequences using CLC Sequence Viewer 7.8.1 (CLC Bio, Aarhus, Denmark). Careful manual checking of chromatograms did show double peaks for herpesviruses found in *R. aegyptiacus*, thus we retained only the mono-infected samples for the phylogenetic analyses. Phylogenetic trees were constructed using BEAST v.1.8.4. [11], using a GTR+I+G substitution model, a strict clock and a constant population size coalescent tree prior. Analyses were run for 100 x10^6 (for viruses) and 300 x 10^6 (for *Leptospira*) generations, sampling every 1,000 generations, with the initial 10% discarded as burn-in. TRACER v.1.635 was then used to verify that the effective sample size of each parameter was higher than 200. The sampled posterior trees were summarized using TREEANNOTATOR v.1.8.4 to generate a maximum clade credibility tree. Sequences produced in this study have been submitted to GenBank database under accession numbers MG680317-MG608402.

Table S1. Summary of statistical models used to analyse infection dynamic and microbiota diversity. N gives the number of samples included in each analysis. Full model includes all the tested variables. “k” is the number of model’s coefficients.

	Variable of interest	Model nb.	N	Full model	k	Significant variables
**Infection dynamics**	*Leptospira* shedding prevalence in *M. natalensis*	GLM_1	73	Session+Sex+Repro+Age	6	Age
	Adenovirus shedding prevalence in *M. natalensis*	GLM_2	90	Session+Sex+Repro+Age	6	Session
	Herpesvirus shedding prevalence in *M. natalensis*	GLM_3	103	Session+Sex+Repro+Age	6	Session+Age
	Herpesvirus shedding prevalence in *R. aegyptiacus*	GLM_4	276	Session+Sex+Repro+Age	7	Session+Age+ Repro
**Microbiota diversity**	Inverse Simpson index in urine in *M. natalensis*	GLM_5	73	Session+Sex+Repro+Age +Lepto	7	Lepto
	Inverse Simpson index in faeces in *M. natalensis*	GLM_6	90	Session+Sex+Repro+Age +AdV	7	Sex
	Inverse Simpson index in saliva in *M. natalensis*	GLM_7	103	Session+Sex+Repro+Age +Herpes	7	Session
	Inverse Simpson index in saliva in *R. aegyptiacus*	GLM_8	276	Session+Sex+Repro+Age +Herpes	8	Session+Sex

Table S2. Model selection tables. Output were obtained with the MuMin package in R (dredge function) for the eight GLMs analyzed (in Table S1). Selected models (after verification of the effect of each variable) are highlighted in red.

Infection dynamics

GLM_1 - Candidate models	*k*	logLik	AIC_c	ΔAIC	weight
Age	2	-32.257	68.7	0.00	0.317
Repro + Age	3	-31.594	69.5	0.85	0.207
Age + Sex	3	-31.996	70.3	1.66	0.139
Age + Repro + Sex	4	-31.103	70.8	2.11	0.110
Session + Age	4	-31.711	72.0	3.33	0.060
Session + Age + Repro	5	-30.994	72.9	4.20	0.039
null	1	-35.682	73.4	4.74	0.030
Session + Age + Sex	5	-31.466	73.8	5.14	0.024
Session + Age + Sex + Repro	6	-30.535	74.3	5.66	0.019
Session	3	-34.407	75.2	6.48	0.012
Repro	2	-35.577	75.3	6.64	0.011
Sex	2	-35.682	75.5	6.85	0.010
Sex + Repro	3	-34.789	75.9	7.24	0.008
Session + Sex	4	-34.359	77.3	8.62	0.004
Session + Repro	4	-34.367	77.3	8.64	0.004
Session + Repro + Sex	5	-33.375	77.6	8.96	0.004

GLM_2 - Candidate models	*k*	logLik	AIC_c	ΔAIC	weight
Session + Sex	4	-21.747	52.0	0.00	0.251
Session + Sex + Repro	5	-20.791	52.3	0.33	0.212
Session	3	-23.200	52.7	0.71	0.175
Session + Repro	4	-22.512	53.5	1.53	0.117
Session + Sex + Age	5	-21.747	54.2	2.24	0.082
Session + Sex + Age + Repro	6	-20.791	54.6	2.63	0.067
Session + Age	4	-23.200	54.9	2.91	0.059
Session + Age + Repro	5	-22.512	55.7	3.77	0.038
Age	2	-54.266	112.7	60.71	0.000
null	1	-55.799	113.6	61.68	0.000
Age + Sex	3	-53.842	114.0	62.00	0.000
Age + Repro	3	-53.969	114.2	62.25	0.000
Sex	2	-55.502	115.1	63.18	0.000
Age + Repro + Sex	4	-53.510	115.5	63.53	0.000
Repro	2	-55.680	115.5	63.53	0.000
Repro + Sex	3	-54.847	116.0	64.01	0.000

GLM$_3$ - Candidate models	*k*	logLik	AIC$_c$	ΔAIC	weight
Session + Age	4	-61.374	131.2	0.00	0.380
Session + Age + Sex	5	-60.760	132.1	0.98	0.232
Session + Age + Repro	5	-61.113	132.8	1.69	0.163
Session + Age + Repro + Sex	6	-60.760	134.4	3.24	0.075
Sex + Age	3	-64.417	135.1	3.92	0.054
Age	2	-65.798	165.7	4.56	0.039
Age + Repro	3	-64.957	136.2	5.00	0.031
Age + Repro + Sex	4	-64.417	137.2	6.09	0.018
Session	3	-67.459	141.2	10.00	0.003
Session + Repro	4	-66.736	141.9	10.72	0.002
Session + Sex	4	-67.051	142.5	11.35	0.001
Session + Sex + Repro	5	-66.736	144.1	12.93	0.001
null	1	-71.156	144.4	13.20	0.001
Sex	2	-70.975	146.1	14.91	0.000
Repro	2	-715155	146.4	15.27	0.000
Repro + Sex	3	-70.721	147.7	16.53	0.000

GLM$_4$ - Candidate models	*k*	logLik	AIC$_c$	ΔAIC	weight
Session + Age + Repro	6	-161.889	336.1	0.00	0.473
Session + Repro	5	-163.845	337.9	1.82	0.190
Session + Age + Repro + Sex	7	-161.858	338.1	2.04	0.170
Session + Repro + Sex	6	-163.772	339.9	3.77	0.072
Session + Age	5	-164.903	340.0	3.94	0.066
Session + Age + Sex	6	-164.724	341.8	5.67	0.028
Session	4	-170.214	348.6	12.48	0.001
Session + Sex	5	-169.854	349.9	13.84	0.000
Age	2	-180.261	364.6	13.84	0.000
Repro + Age	3	-180.071	366.2	30.14	0.000
Age + Sex	3	-180.256	366.6	30.51	0.000
Repro	2	-181.788	367.6	31.53	0.000
Repro + Age + Sex	4	-180.059	368.3	32.17	0.000
Repro + Sex	3	-181.783	369.7	33.56	0.000
null	1	-183.820	369.7	33.56	0.000
Sex	2	-183.805	371.7	35.56	0.000

Microbiota diversity

GLM5 - Candidate models	*k*	logLik	AIC$_c$	ΔAIC	weight
Lepto	3	-247.444	501.2	0.00	0.242
Lepto + Sex	4	-246.824	502.2	1.00	0.147
Lepto + Repro	4	-247.045	502.7	1.44	0.118
Lepto + Age	4	-247.441	503.5	2.23	0.079
Lepto + Session + Sex	6	-245.386	504.0	2.81	0.059
Lepto + Repro + Sex	5	-246.800	504.5	3.26	0.047
Lepto + Age + Sex	5	-246.806	504.5	3.27	0.047
Lepto + Repro	5	-246.845	504.6	3.35	0.045
Lepto + Repro + Age	5	-247.036	505.0	3.73	0.037
Lepto + Session + Repro	6	-245.924	505.1	3.88	0.035
Lepto + Session + Sex + Age	7	-245.132	506.0	4.75	0.022
Lepto + Session + Sex + Repro	7	-245.345	506.4	5.18	0.018
Lepto + Sex + Repro + Age	6	-246.784	506.8	5.60	0.015
Lepto + Session + Age	6	-246.841	507.0	5.72	0.014
null	2	-251.548	507.3	6.03	0.012
Lepto + Session + Repro + Age	7	-245.779	507.3	6.04	0.012
Sex	3	-250.991	508.3	6.04	0.012
Age	3	-251.010	508.4	7.13	0.007
Lepto + Session + Repro + Age + Sex	8	-245.096	508.4	7.20	0.007
Repro	3	-251.307	509.0	7.73	0.005
Session + Sex	5	-249.148	509.2	7.95	0.005
Session	4	-250.619	509.8	8.59	0.003
Age + Sex	4	-250.650	509.9	8.65	0.003
Repro + Sex	4	-250.846	510.3	9.04	0.003
Repro + Age	4	-250.885	510.4	9.12	0.003
Session + Repro	5	-249.899	510.7	9.46	0.002
Session + Repro + Sex	6	-248.957	511.2	9.95	0.002
Session + Age + Sex	6	-249.122	511.5	10.28	0.001
Session + Age	5	-250.327	511.5	10.31	0.001
Repro + Age + Sex	5	-250.504	511.9	10.67	0.001
Session + Repro + Age	6	-249.797	512.9	11.63	0.001
Session + Repro + Age + Sex	7	-248.934	513.6	12.35	0.001

**GLM_6 - Candidate models**	***k***	**logLik**	**AIC_c**	**ΔAIC**	**weight**
Sex	3	-185.315	376.9	0.00	0.255
Repro	3	-185.955	378.2	1.28	0.134
Sex + Repro	4	-185.284	379.0	2.13	0.088
Sex + Age	4	-185.297	379.1	2.16	0.087
Sex + AdV	4	-185.314	379.1	2.19	0.085
Repro + AdV	4	-185.945	380.4	3.45	0.045
Repro + Age	4	-185.952	380.4	3.470	0.045
Session + Sex	5	-185.030	380.8	3.86	0.037
Sex + Repro + Age	5	-185.240	381.2	4.29	0.030
Sex + Repro + AdV	5	-185.284	381.3	4.37	0.029
Sex + AdV + Age	5	-185.295	381.3	4.39	0.028
Session + Repro	5	-185.703	382.1	5.21	0.019
Session + Sex + Age	6	-184.709	382.4	5.52	0.016
Repro + AdV + Age	5	-185.943	382.6	5.69	0.015
Session + Sex + Age	6	-184.989	383.0	6.08	0.012
Session + Sex + Repro	6	-184.997	383.0	6.10	0.012
Sex + Repro + AdV + Age	6	-185.239	383.5	6.58	0.009
Session + Repro + Age	6	-185.545	384.1	7.19	0.007
null	2	-190.075	384.3	7.38	0.006
Session + Repro + Age	6	-185.696	384.4	7.38	0.006
Session + Repro + Sex + AdV	7	-184.635	384.6	7.73	0.005
Session + Sex + Age + AdV	7	-184.685	384.7	7.83	0.005
Session	4	-188.212	384.9	7.99	0.005
Session + Repro + Sex + Age	7	-184.905	385.2	8.27	0.004
Session + Age	5	-187.544	385.8	8.89	0.003
Age	3	-189.956	386.2	9.28	0.002
AdV	3	-190.015	386.3	9.40	0.002
Session + Repro + Age + AdV	7	-185.543	386.5	9.54	0.002
Session + Repro + Age + Sex + AdV	8	-184.553	386.9	9.97	0.002
Session + AdV	5	-188.190	387.1	10.19	0.002
Session + Age + AdV	6	-187.521	388.1	11.15	0.001
Age + AdV	4	-187.521	388.2	11.29	0.001

GLM$_7$ - Candidate models	*k*	logLik	AIC$_c$	ΔAIC	weight
Session	4	-333.571	675.5	0.00	0.259
Session + Herpes	5	-333.202	677.0	1.47	0.124
Session + Repro	5	-333.277	677.2	1.62	0.115
Session + Sex	5	-333.534	677.7	2.14	0.089
Session + Age	5	-333.566	677.8	2.20	0.086
Session + Repro + Herpes	6	-333.813	678.5	2.95	0.059
Session + Age + Herpes	6	-333.115	679.1	3.56	0.044
Session + Sex + Herpes	6	-333.140	679.2	3.61	0.043
Session + Repro + Sex	6	-333.182	679.2	3.69	0.041
Session + Repro + Age	6	-333.209	679.3	3.74	0.040
Session + Sex + Age	6	-333.533	679.9	4.39	0.029
Session + Sex + Repro + Herpes	7	-332.719	380.6	5.07	0.021
Session + Herpes + Repro + Age	7	-332.812	680.8	5.25	0.019
Session + Herpes + Age + Sex	7	-333.104	681.4	5.84	0.014
Session + Repro + Age + Sex	7	-333.145	681.5	5.92	0.013
Session + Repro + Age + Sex + Herpes	8	-332.718	683.0	7.42	0.006
Sex + Age	4	-344.167	696.7	21.19	0.000
Sex + Age + Herpes	5	-344.088	698.8	23.25	0.000
Sex + Age + Repro	5	-344.132	698.9	23.33	0.000
Sex	3	-346.808	699.9	24.31	0.000
Sex + Age + Repro + Herpes	6	-344.054	701.0	25.43	0.000
Sex + Repro	4	-346.506	701.4	25.87	0.000
Sex + Herpes	4	-346.722	701.9	26.30	0.000
Age + Repro	4	-347.049	702.5	26.96	0.000
Sex + Repro + Herpes	5	-346.440	703.5	27.95	0.000
Repro + Herpes + Age	5	-347.038	704.7	29.14	0.000
Repro	3	-349.295	704.8	29.28	0.000
null	2	-350.845	705.8	30.26	0.000
Age	3	-350.007	706.3	30.71	0.000
Herpes + Repro	4	-349.134	706.7	31.13	0.000
Herpes	3	-350.685	707.6	32.06	0.000
Herpes + Age	4	-349.992	708.4	32.84	0.000

**GLM_8 - Candidate models**	***k***	**logLik**	**AIC_c**	**ΔAIC**	**weight**
Session + Sex	6	-259.027	530.4	0.00	0.286
Session + Sex + Herpes	7	-258.952	532.3	1.96	0.107
Session + Sex + Age	7	-258.970	532.4	1.99	0.106
Session + Sex + Repro	7	-259.026	532.5	2.10	0.100
Session	5	-261.168	532.6	2.19	0.095
Session + Sex + Herpes + Age	8	-258.915	534.4	4.00	0.039
Session + Sex + Herpes + Repro	8	-258.951	534.4	4.08	0.037
Session + Sex + Age + Repro	8	-258.958	534.5	4.09	0.037
Session + Herpes	6	-261.129	534.6	4.20	0.035
Session + Age	6	-261.160	534.6	4.27	0.034
Session + Repro	6	-261.166	534.6	4.28	0.034
Session + Sex + Age + Herpes + Repro	9	-258.895	536.5	6.10	0.014
Session + Herpes + Repro	7	-261.121	536.7	6.29	0.012
Session + Herpes + Age	7	-261.126	536.7	6.30	0.012
Session + Repro + Age	7	-261.149	536.7	6.35	0.012
Sex	3	-265.718	537.5	7.16	0.008
Sex + Age	4	-265.234	538.6	8.25	0.005
Session + Herpes + Repro + Age	8	-261.109	538.8	8.39	0.004
null	2	-267.565	539.2	8.81	0.003
Sex + Repro	4	-265.608	539.4	9.00	0.003
Sex + Herpes	4	-265.656	539.5	9.09	0.003
Age	3	-266.767	539.6	9.26	0.003
Sex + Herpes + Age	5	-265.115	540.5	10.09	0.002
Sex + Age + Repro	5	-265.211	540.6	10.28	0.002
Repro	3	-267.301	540.7	10.33	0.002
Herpes	3	-267.511	541.1	10.74	0.001
Sex + Herpes + Repro	5	-265.524	541.3	10.90	0.001
Herpes + Age	4	-266.640	541.4	11.06	0.001
Repro + Age	4	-266.759	541.7	11.30	0.001
Sex + Herpes + Repro + Age	6	-265.096	542.5	12.14	0.001
Herpes + Repro	4	-267.213	542.6	12.21	0.001
Herpes + Repro + Age	5	-266.635	543.5	13.13	0.000

Table S3. Details of the number of bat samples collected at different sample periods.

	September	October/November	January	April	Total
M. natalensis					
Oral	19	36	48	0	103
Faeces	23	39	28	0	90
Urine	4	45	24	0	73
Subtotal	*46*	*120*	*100*	*0*	*266*
R. aegyptiacus					
Oral	47	95	38	96	276
**Total**	**93**	**215**	**138**	**96**	**542**

Figure S1. Variation of microbiota diversity

(*a*) Effect of sampling session

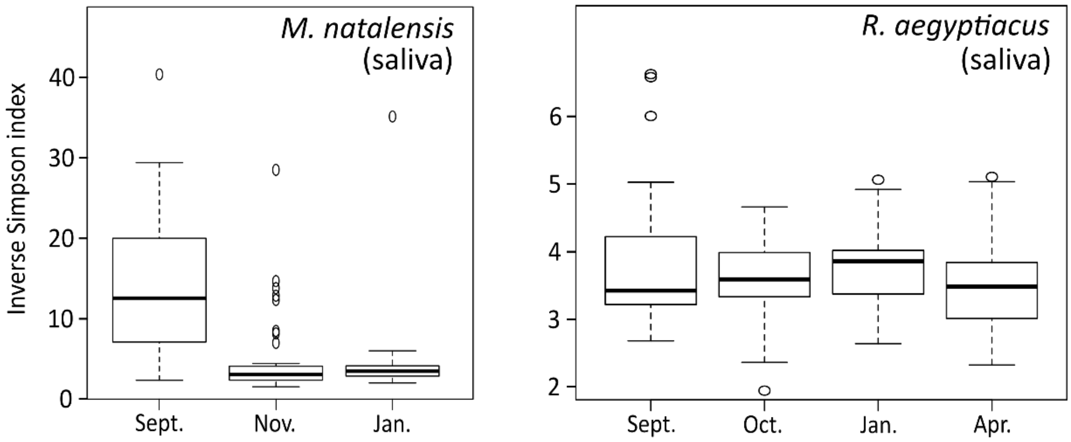

(*b*) Effect of sex

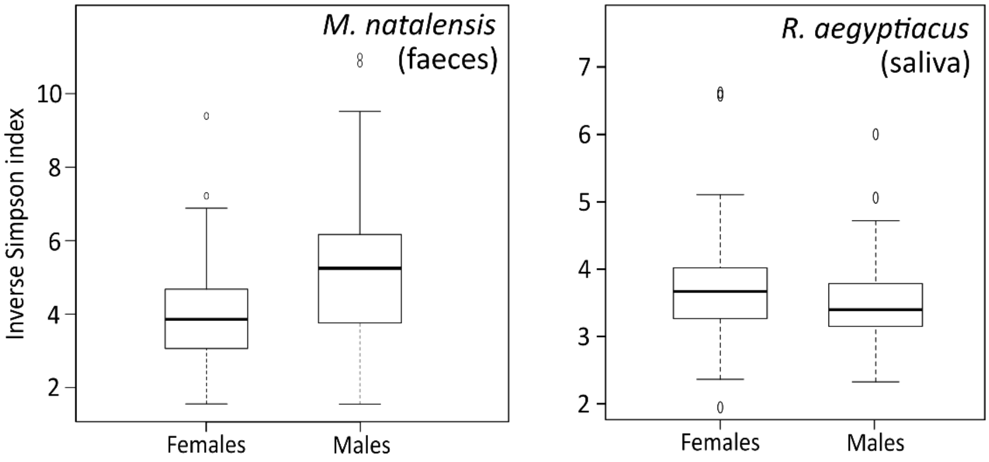

References

1. Yuan S, Cohen DB, Ravel J, Abdo Z, Forney LJ. 2012 Evaluation of Methods for the Extraction and Purification of DNA from the Human Microbiome. *PLoS One* **7**, e33865. (doi:10.1371/journal.pone.0033865)
2. Pearce MM *et al.* 2014 The Female Urinary Microbiome : a Comparison of Women with and without Urgency Urinary Incontinence. *MBio* **5**, 1–12. (doi:10.1128/mBio.01283-14.Editor)
3. Dietrich M, Kearney T, Seamark ECJ, Markotter W. 2017 The excreted microbiota of bats : evidence of niche specialisation based on multiple body habitats. *FEMS Microbiol. Lett.* **364**, fnw284. (doi:10.1093/femsle/fnw284)
4. Kozich JJ, Westcott SL, Baxter NT, Highlander SK, Schloss PD. 2013 Development of a Dual-Index Sequencing Strategy and Curation Pipeline for Analyzing Amplicon Sequence Data on the MiSeq. *Appl. Environ. Microbiol.* **79**, 5112–5120. (doi:10.1128/AEM.01043-13)
5. Schloss PD *et al.* 2009 Introducing mothur : Open-Source, Platform-Independent, Community-Supported Software for Describing and Comparing Microbial Communities. *Appl. Environ. Microbiol.* **75**, 7537–7541. (doi:10.1128/AEM.01541-09)
6. Edgar RC, Haas BJ, Clemente JC, Quince C, Knight R. 2011 UCHIME improves sensitivity and speed of chimera detection. *Bioinformatics* **27**, 2194–2200. (doi:10.1093/bioinformatics/btr381)
7. VanDevanter DR, Warrener P, Bennett L, Schultz ER, Coulter S, Garber RL, Rose TM, Black J. 1996 Detection and Analysis of Diverse Herpesviral Species by Consensus Primer PCR. *J. Clin. Microbiol.* **34**, 1666–1671.
8. Li Y *et al.* 2010 Host Range, Prevalence, and Genetic Diversity of Adenoviruses in Bats. *J. Virol.* **84**, 3889. (doi:10.1128/JVI.02497-09)
9. Smythe LD, Smith IL, Smith GA, Dohnt MF, Symonds ML, Barnett LJ, McKay DB. 2002 A quantitative PCR (TaqMan) assay for pathogenic Leptospira spp. *BMC Infect. Dis.* **2**, 13.
10. Dietrich M, Wilkinson DA, Soarimalala V, Goodman SM, Dellagi K, Tortosa P. 2014 Diversification of an emerging pathogen in a biodiversity hotspot: Leptospira in endemic small mammals of Madagascar. *Mol. Ecol.* **23**, 2783–96. (doi:10.1111/mec.12777)
11. Guindon S, Gascuel O. 2003 A simple, fast, and accurate algorithm to estimate large phylogenies by maximum likelihood. *Syst. Biol.* **52**, 696–704. (doi:10.1080/10635150390235520)
12. Crawley MJ. 2007 *The R Book*. John Wiley. Chichester, UK.
